# Examining the sources of evidence in e-cigarette policy recommendations: A citation network analysis of international public health recommendations

**DOI:** 10.1371/journal.pone.0255604

**Published:** 2021-08-04

**Authors:** Marissa J. Smith, Andrew J. Baxter, Kathryn Skivington, Mark McCann, Shona Hilton, Srinivasa Vittal Katikireddi

**Affiliations:** MRC/CSO Social and Public Health Sciences Unit, University of Glasgow, Glasgow, United Kingdom; University of Toronto, CANADA

## Abstract

**Background:**

Public health policies and recommendations aim to be informed by the best available evidence. Evidence underpinning e-cigarettes policy recommendations has been necessarily limited due to the novelty of the technology and the lack of long-term epidemiological studies and trials. Some public health bodies have actively encouraged e-cigarette use whilst others have raised concerns over introducing new health risks and renormalising tobacco smoking. Using citation network analysis we investigated the author conflicts of interest and study funding statements within sources of evidence used by public health bodies when making recommendations about e-cigarette policy.

**Methods:**

We conducted citation network analysis of public health recommendation documents across four purposively selected diverse jurisdictions: WHO, UK, Australia, and USA. We extracted all citations from 15 public health recommendation documents, with more detailed data collected for influential citations (used in 3+ recommendation documents). We analysed the relationships between the sources of evidence used across jurisdictions using block modelling to determine if similar groups of documents were used across different jurisdictions. We assessed the frequency and nature of conflicts of interest.

**Results:**

1700 unique citations were included across the 15 public health recommendation documents, with zero to 923 citations per document (median = 63, IQR = 7.5–132). The evidence base underpinning public health recommendations did not systematically differ across jurisdictions. Of the 1700 citations included, the majority were journal articles (n = 1179). Across 1081 journal articles published between 1998–2018, 200 declared a conflict of interest, 288 contained no mention of conflicts of interest, and 593 declared none. Conflicts of interest were reported with tobacco (3%; n = 37 journal articles of 1081), e-cigarette (7%; n = 72), and pharmaceutical companies (12%; n = 127), with such conflicts present even in the most recent years. There were 53 influential citations, the most common study type was basic science research without human subjects (e.g. examination of aerosols and e-liquids) (n = 18) followed by systematic review (n = 10); with randomised control trial being least common (n = 4). Network analysis identified clusters of highly-cited articles with a higher prevalence of conflicts of interest.

**Conclusion:**

Public health bodies across different jurisdictions drew upon similar sources of evidence, despite articulating different policy approaches to e-cigarettes. The evidence drawn upon, including the most influential evidence, contained substantial conflicts of interest (including relationships with e-cigarette and tobacco industries). Processes to explicitly manage conflicts of interest arising from the underlying evidence base may be required when developing public health recommendations.

## Introduction

Public health policies and recommendations aim to be informed by the best available evidence [1, 2]. The quality of evidence is a key element of decisions making and several frameworks, such as GRADE (Grades of Recommendation, Assessment, Development and Evaluation), have been developed to assist in the assessing of evidence [3]. Public health policies and recommendation documents can be characterised as documents or statements that contain recommendation(s) for health practice, public health, or health policy [4]. It is generally agreed that the process for developing public health policies should be transparent and lead to impartial decisions that improve health, based on the best available evidence [5].

Electronic cigarettes (e-cigarettes, also known as Electronic Nicotine Delivery Systems or ENDS) are an important case study to understand how evidence informs public health recommendation documents, given the rapid development of the evidence base. Numerous public health bodies across the world have released reports, recommendation documents, and statements on their position on e-cigarettes. A range of policy/regulatory and public health approaches towards e-cigarettes products has been pursued across the globe from being completely prohibited to being regulated as consumer products, tobacco products, or medicinal devices [6, 7]. Different policy/regulatory and public health approaches may be being pursued due to the different sources of evidence drawn upon [7]. Alternatively, it is possible that decision-makers may be drawing on a similar evidence base, however, still making different recommendations either due to prioritisation of different goals or adaptation to their jurisdiction [8]. Network analysis can help identify whether recommendation documents draw on similar or different sources of evidence, helping to explore if there are underlying structures in how research evidence is used to developing recommendation documents.

The potential for conflicts of interest (COI) is present in all areas of public health. It occurs when “a set of circumstances that creates a risk that an individual’s professional judgement or actions regarding a primary interest (e.g. validity of research) is, or could be, impaired or influenced by a secondary interest (e.g. financial gain)” [9]. COI may threaten the integrity of scientific investigations, undermine the evidence base, and risk threatening the trustworthiness of recommendations [9]. An important concern is that COI amongst policymakers or guideline developers may act as a potential source of bias in the development of public health recommendations [1, 10]. COI may also be present among the evidence base which policy and decision-makers draw upon and this may lead to recommendations being distorted to favour a secondary interest. While both of these aspects of COI are important, our focus in this paper is on the latter. The impact of COI within the underlying evidence base could potentially differ across jurisdictions too. If some jurisdictions draw on different sources of evidence than others, this may mean that secondary interests may have a greater effect in some areas than others. Alternatively, if some key papers influence recommendation documents across all jurisdictions, the presence of COI in these papers may favour secondary interests in all jurisdictions. Thus, the management of COI in all stages of the process is essential for the development of high-quality recommendations [11]. Author COI may arise from influence on authors through financial payments from companies to researchers for consulting, advisory roles, speaking etc. and are reported in COI statements [9]. Although receipt of study funding (financial support from companies for conducting the research and is usually paid to the institution) is not always recorded as a COI for the author(s), it is associated with other concerns including agenda setting and sponsorship bias [12–15]. The tobacco industry, in particular, has a long history of reporting industry favourable results [16], with Article 5.3 of the World Health Organisation (WHO) Framework Convention on Tobacco Control focusing on limiting its influence on public health policy. The presence of study funding from a commercial entity and/or COI among the authors of articles forming the evidence base may induce bias and contribute to differences in recommendations concerning e-cigarettes.

This study investigates the sources of evidence in relation to the types of evidence used by public health bodies across four diverse jurisdictions when making e-cigarette policy recommendations.

Further, it examines the author conflicts of interest and study funding statements within these sources, to deepen our understanding of the diffusion of industry funded and industry-supported evidence in public health recommendation documents.

## Methods

### Selection of study contexts

We purposively selected four different influential jurisdictions, considered important for setting the agenda on policy recommendation for e-cigarettes policy: WHO, UK (Scotland, England, Wales, and Northern Ireland), Australia, and USA. These jurisdictions were selected to reflect the different approaches towards e-cigarette regulation [17]. The UK has adopted a ‘harm reduction’ approach towards e-cigarettes, proposing that smokers should be encouraged to switch to e-cigarettes [16, 18]. In contrast WHO, Australia, and USA have adopted a ‘precautionary’ approach, arguing that smokers should be encouraged to quit smoking and not switch to e-cigarettes [16]. The contrasting policy/regulatory and public health approaches provides an opportunity to investigate the sources of evidence drawn upon by public health bodies when developing e-cigarette recommendations.

Sub-national level bodies within the UK were included in the sample to investigate the diversity within a jurisdiction. The UK has four public health systems and they correspond to its four different political systems. Scotland, Wales, and Northern Ireland each have an autonomous legislature that makes health policy while the UK Government directly runs England’s National Health Service (NHS) [19, 20]. This therefore makes the UK an interesting and complex case to examine. However, it was not feasible to include sub-national level bodies within Australia and USA.

### Identification of sample

Within each of the chosen contexts, we identified public health bodies that had produced public health recommendation documents, position papers, or policy statements on e-cigarettes that included recommendations for health practice, public health and/or health policy. A ‘public health body’ was defined as an organisation whose aims stated, or whose role within local/national/international policy is to protect and improve the health of a population. Several public health bodies had been identified during the literature review stage of the research and through correspondence with experts in the field. Additional public health bodies were identified using online searching. The online search for the public health bodies and recommendation documents was conducted between July and August 2019. As the literature surrounding e-cigarettes is continuously evolving, another online search was conducted in December 2019 to ensure no documents had been missed from the sample. Websites of public health bodies were searched for any publicly available documents using the key terms “e-cigarettes”, “electronic cigarettes”, “e-liquids”, and “tobacco”. Citation lists within the identified documents were examined for additional relevant recommendation documents, position, or policy statements. The criteria for sample inclusion is shown in Table 1.

**Table 1:** Criteria for including documents in the sample.

INCLUSION	EXCLUSION
Documents had to be published as a report or in similar document form	Webpages, fact sheets, research articles, and media releases
Published between 2014–2019	Published before 2014 or after 2019
Published in English	Not in English
Published by a public health body	Medical organisations, patient organisations, health charities and government policy
Policy recommendations relating to e-cigarettes had to be made (e.g., regarding advertising and promotion of e-cigarette products)	Detailed only research recommendations
Provided at least two policy recommendations on e-cigarettes	Provided fewer than two policy recommendations on e-cigarettes

Through snowballing from websites, policy documents, and personal contacts, a list of relevant experts within each jurisdiction was compiled. These experts were emailed with a list of the documents making up the sample and asked to provide details of any recommendation documents, positions, or policy statement documents they believed to be influential that were not included in the original sample. The search strategy is presented in S1 Appendix.

A total of 15 documents across 10 public health bodies (eight from the UK, two from Australia, three from the USA, and two from WHO) met the inclusion criteria for further analysis.

### Citation network analysis

Citation analysis measures the importance or impact of an author, an article, or a publication by counting times cited in other works, and network analysis can be used to study patterns of connections between documents, where a citation is considered a link between documents in the network [21]. We extracted all citations from the 15 documents into an Excel spreadsheet, giving each cited document a unique identifier (across all recommendation documents). These were imported into R (v 3.6.1; R Core Team, 2019), tidied, and deduplicated before constructing a two-mode adjacency matrix charting unique citations across recommendation documents [22]. Recommendation documents with no citations were removed (n = 1). We constructed bipartite network graphs using R and igraph [23]. We plotted recommendation documents and references as separate classes of nodes; edges denoted citation of a reference in a recommendation document. We used the Fruchterman-Reingold force-directed algorithm for placing nodes to visualise closeness of recommendation-document connection through the number of shared references. Our initial network graph plotted all citations across 14 documents, coloured by the number of times cited. To visualise high-impact citations, we selected those cited across three or more of the recommendation documents for more detailed analysis. This criterion was selected based on a pragmatic decision between the authors following exploratory visualisation of the full network and it was determined that those cited across three or more recommendations documents was more manageable for further analysis. From these, we manually extracted study type and COI and funding statements (this included authors’ financial ties and commercial funding). Within the literature COI and study funding are sometimes reported separately; receipt of study funding from a commercial entity is not always considered a COI for the author(s) [15], but the International Committee on Medical Journal Editors recommend declaring such funding as a potential COI [24]. We classify both author declarations of interest and study funding statements as COI, including declarations of financial relationships with commercial entities and industry influence.

We assessed COI statements in the publications and checked supplementary material (such as ICMJE forms) when referenced and available. If an author did not provide a COI statement in the manuscript and did not refer to supplementary material elsewhere, this was classified as ‘no mention’ of COI. We constructed three further graphs of high-impact citations, colouring by the number of times cited, type of publication, and type of COI. Publication types were classed as classed basic science research without human subjects (e.g. examination of aerosols and e-liquids), systematic review (SR), non-systematic review, longitudinal observational study, cross-sectional and randomised control trial (RCT).

We conducted in-depth analysis of the 53 influential citations to determine if the interpretation of the citations varied across recommendation documents. This was done by examining the surrounding text of when the reference was cited within the recommendation document. Where COI were declared in a cited document, we recorded whether this was assessed in the recommendation document. The primary coder was MS and a random sample of 20% was double coded by KS.

### Retrieval of conflicts of interest statements

To retrieve information and categorise each of the citations, we built a Shiny interactive web app (S2 Appendix). Shiny is an R package that allows the creation of interactive web applications, combining the statistical power of R and the interactivity of the modern web [25]. It is an efficient alternative to spreadsheets, printed visualisations and saves space and time in the construction, automation and distribution of data visualisation and statistical analysis [25, 26]. Citations were categorised by type to provide an insight into the different types of evidence being drawn upon by public health bodies when making e-cigarette recommendations. Analysis focused on cited journal article texts. Journal articles were defined as journal publications consisting of an academic study or information (e.g., essay) concerning a particular topic/discipline. Cited journal article texts were searched for COI then extracted texts were coded manually for the presence of COI types (S2 Appendix). We also checked supplementary material (such as ICMJE forms) if the Shiny app detected the authors’ reference to these. The primary coder was MS and a random sample of 10% was independently double coded by a second author (AB) and there was full agreement on the second coding. A random sample of at least four government/official reports from each of the 14 recommendation documents were screened for COI statements (n = 64). Government/official reports were defined as documents or reports that had been produced by official organisations (e.g., WHO FCTC, regulators). As no COI were found, this category was excluded from further analysis of COI.

### Statistical analysis

To analyse whether recommendation documents drew upon similar evidence, we used bipartite stochastic block modelling to detect clustering within the citation network. The method aims to detect if there are groups of documents that are similar based on their connections to other documents in the citation network. We examined which recommendation documents drew upon similar sources of evidence and created groups of recommendation documents by strength of connection. The clustering of evidence sources was determined by their co-occuring citations in recommendation documents. We fitted a series of block models, with between 1 and 10 blocks (referred to as ‘groups’ for the remainder of the article) of recommendation documents, and between 1 and 15 blocks (referred to as ‘clusters’) of evidence sources [27]. We used log-likelihood to identify the number of blocks that best fitted the structure of the citation network and selected the number of blocks based on model fit, parsimony, and interpretation of the recommendation document membership. We conducted fisher’s exact tests to determine whether the proportion of COI was differently distributed across recommendation groups and reference clusters (as several count values were low).

## Results

A total of 1700 unique citations were included across the 15 public health recommendation documents, with zero to 923 citations per recommendation document (median = 63, IQR = 7.5–132) (Table 2). The NHS Health Scotland 2017 document (NHS HS 2017) did not include any citations and therefore was not included in further analysis.

**Table 2:** Number of citations within each of the 15 selected public health recommendation documents.

Context	Public Health Body	Document	Number of citations in document
International	World Health Organisation	Electronic nicotine delivery systems (2014)	30
		Electronic Nicotine Delivery Systems and Electronic Non-Nicotine Delivery Systems (ENDS/ENNDS) (2016)	89
UK	National Institute for Health and Care Excellence	Stop smoking intervention and services [NG92] (2018)	9
	NHS Health Scotland	Smoke-free prisons and e-cigarettes (2016)	5
		Consensus statement on e-cigarettes (2017)	0
	Public Health England	E-cigarettes: an evidence update (2015)	178
		Use of e-cigarettes in public places and workplaces (2016)	11
		Evidence review of e-cigarettes and heated tobacco products (2018)	404
		Vaping in England: an evidence update (2019)	82
	Public Health Wales	E-cigarettes (Electronic Nicotine Delivery Systems (ENDS)) (2017)	6
Australia	National Health and Medical Research Council	National Health and Medical Research Council CEO Statement: Electronic Cigarettes (E-Cigarettes) (2017)	69
	Public Health Association Australia	E-cigarettes policy position statement (2018)	6
USA	American Public Health Association	Supporting regulation of Electronic Nicotine Delivery Systems (2018)	86
	U.S Department of Health and Human Services	E-Cigarette Use Among Youth and Young Adults: A Report of the Surgeon General (2016)	923
	U.S. Food and Drug Administration	Deeming Tobacco Products To Be Subject to the Federal Food, Drug, and Cosmetic Act, as Amended by the Family Smoking Prevention and Tobacco Control Act; Restrictions on the Sale and Distribution of Tobacco Products and Required Warning Statements for Tobacco Products (2016)	63

The year of publication of the 1700 citations ranged from 1947–2019 (median = 1994, IQR = 1981–2007). Citations were categorised by type; the most common was journal article (n = 1189), followed by Government/official report (n = 267). E-cigarette company press releases (n = 3) and commentaries on papers (n = 1) and were the least common. Please see S1 Table for the other types of citations and S2 Table for the types of citation across the four jurisdictions. Statistical reports were classified as reports that specifically detailed statistics (e.g., the number of youths using e-cigarette products or the number of e-cigarette users, etc.). Of note, there were 15 citations that included COI with tobacco companies. COI associated with tobacco companies were classified when an author stated that they had worked and/or received payment from a tobacco company and/or when research was funded by a tobacco company (e.g., “Tanvir Walee is an employee of Fontem Ventures B.V and Josie Williams is an employee of Imperial Tobacco Group. Girish Sharma, Rebecca Savioz and Claire Martin receive personal fee from Fontem Venture B.V.”) [28].

**Fig 1:**
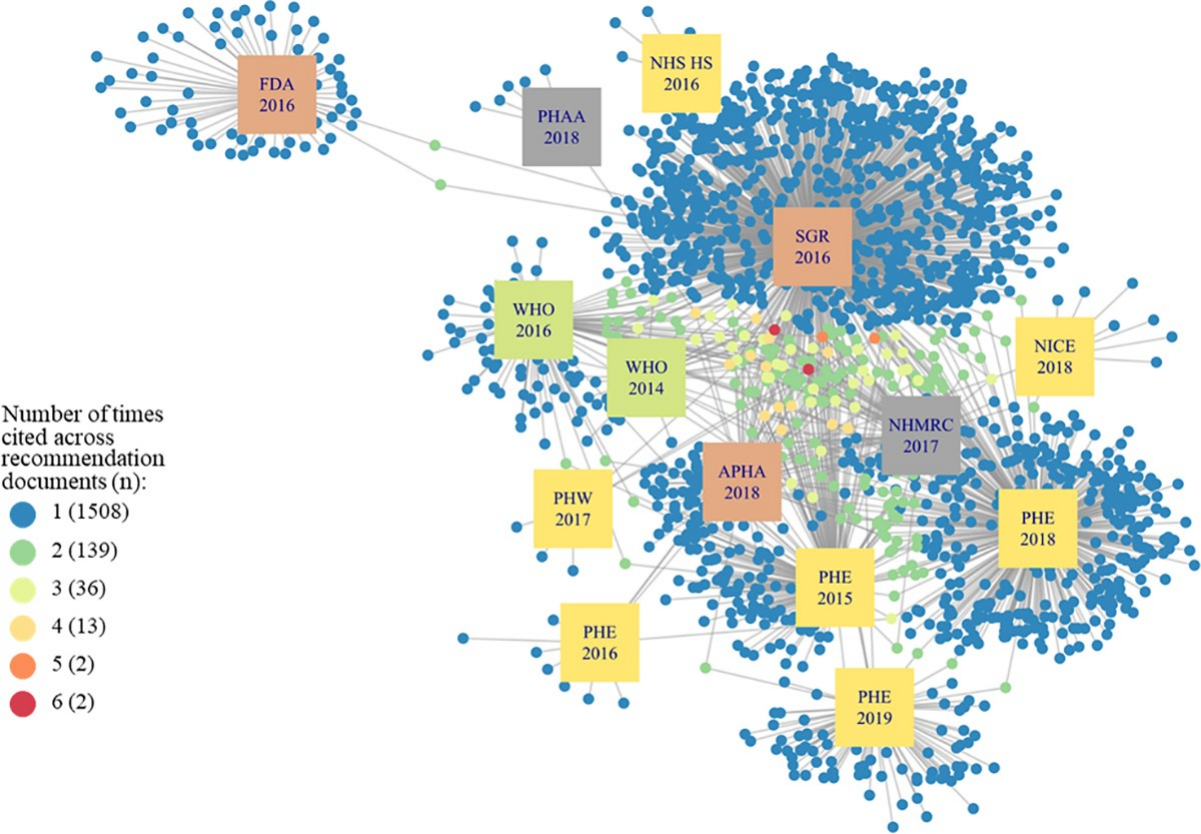
Citation network illustrating the 1700 evidence sources cited in 14 recommendation documents. APHA = American Public Health Association; FDA = U.S. Food and Drug Administration; NHMRC = National Health and Medical Research Council (AUS); NHS HS = NHS Health Scotland; NICE = National Institute for Health and Care Excellence (UK); PHAA = Public Health Association Australia; PHE = Public Health England; PHW = Public Health Wales; SGR = U.S. Department of Health and Human Services: A Report of the Surgeon General.

The network graph in Fig 1 illustrates how citations were cited across the 14 recommendation documents. NHS HS (2017) did not include any citations in the document, therefore, is not shown in Fig 1. Several recommendation documents are clustered in the centre of the graphs, sharing most of their citations with other recommendation documents. The U.S Food and Drug Administration (FDA) 2016 shared few references with other recommendation documents, therefore is distinctly detached from the other documents.

**Fig 2:**
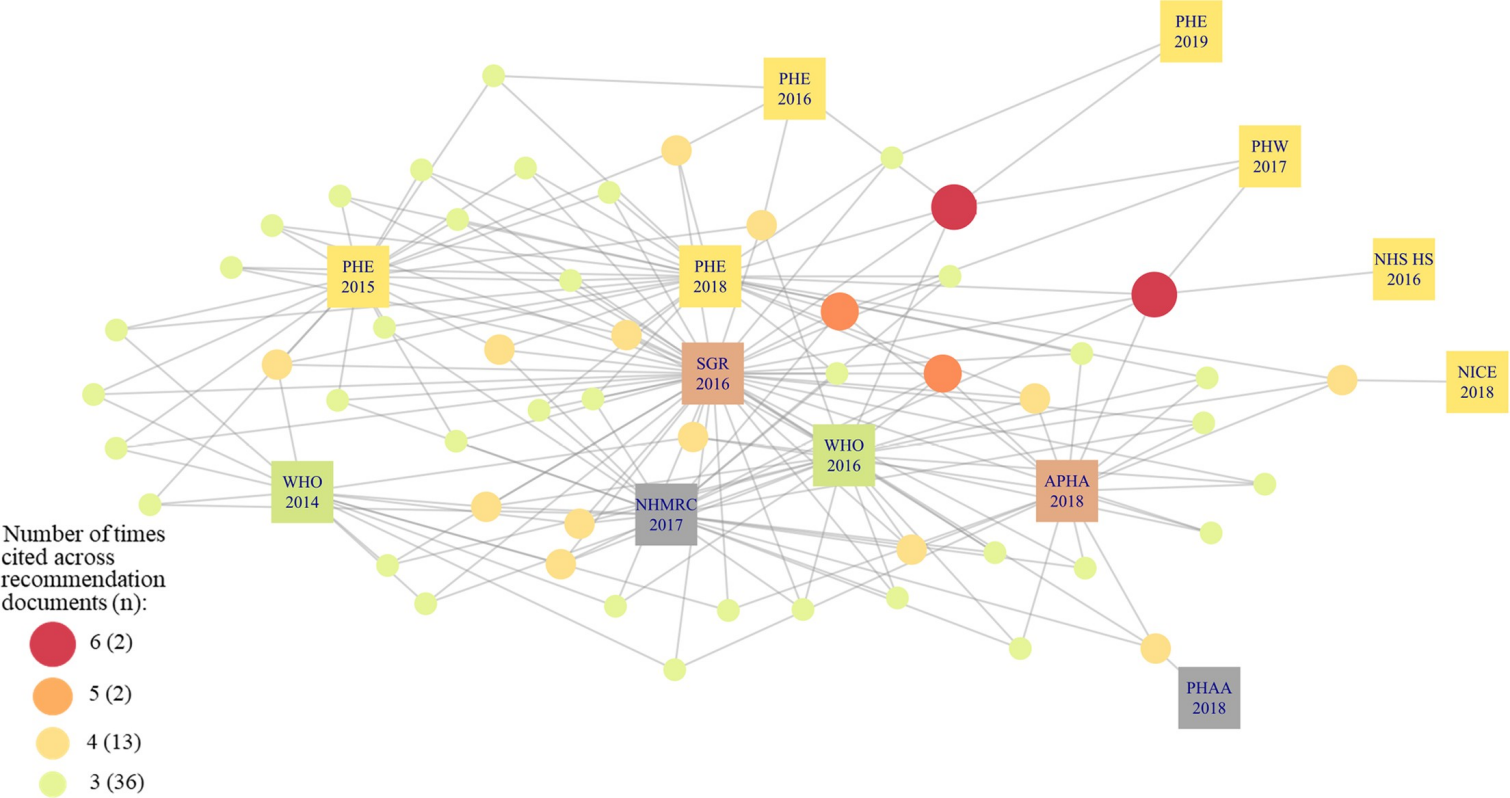
Citation network for the 53 most highly cited citations across 13 recommendation documents.

**Fig 3:**
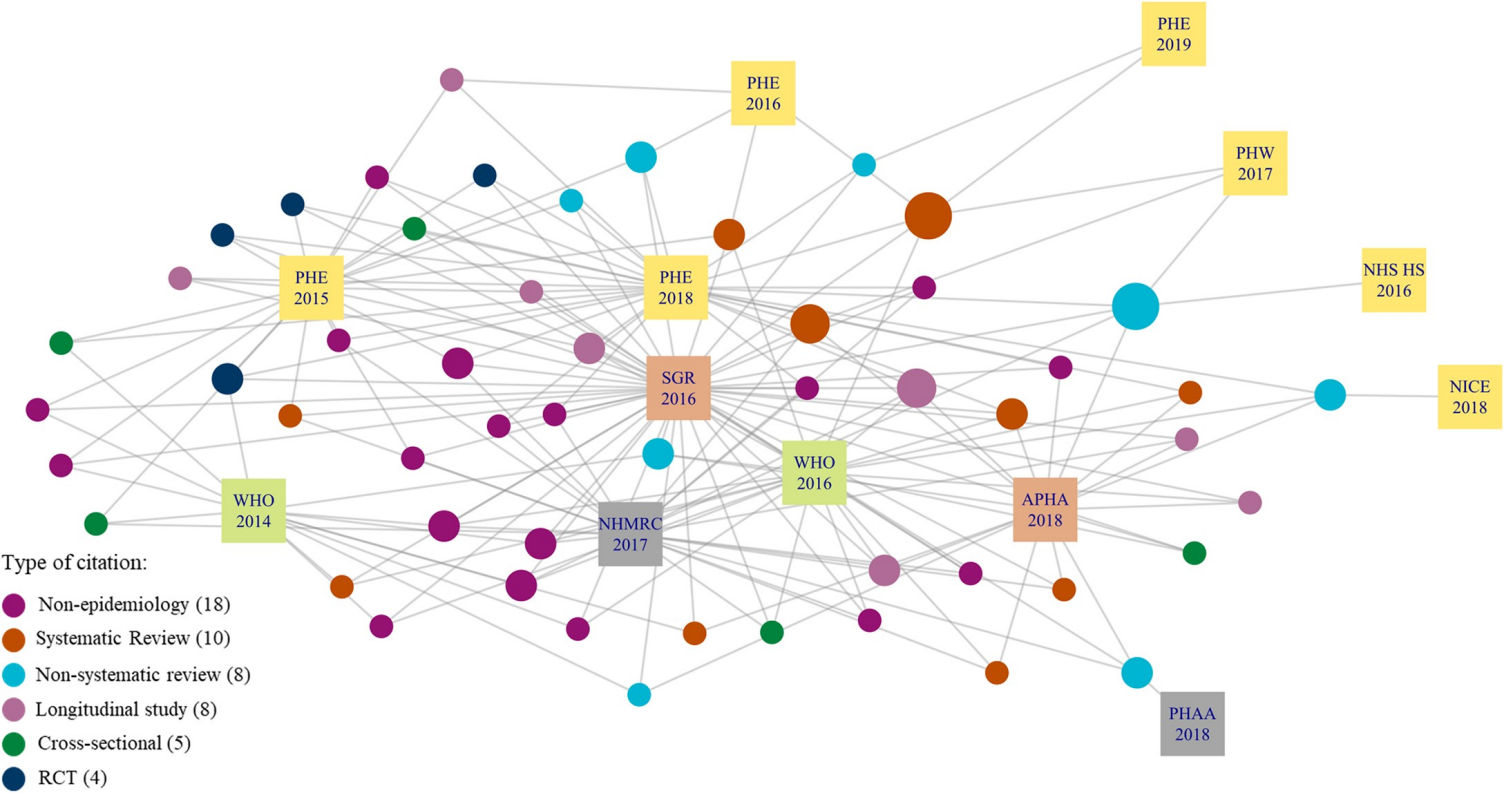
Citation network showing the study design of the 53 highly cited citations across 13 recommendation documents.

**Fig 4:**
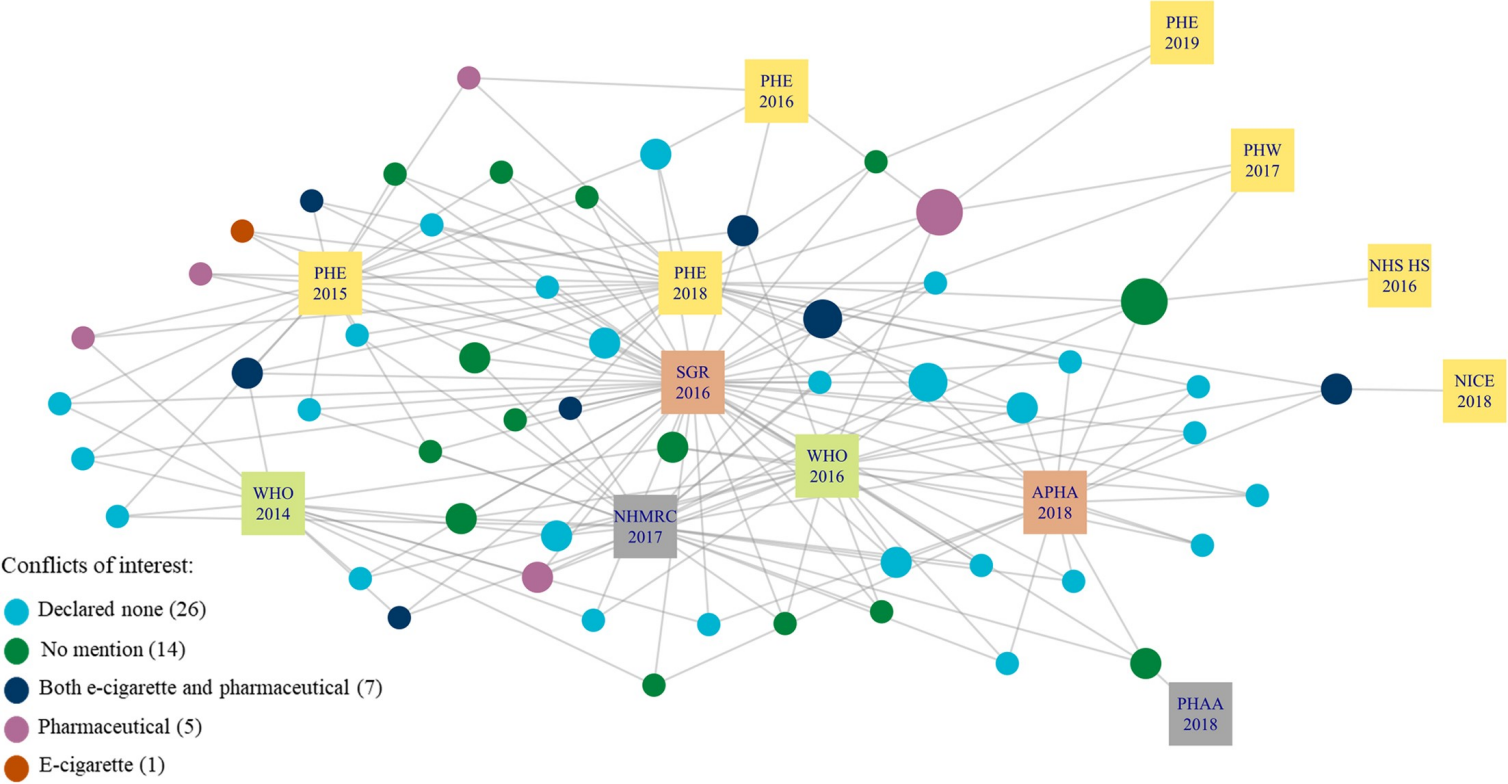
Citation network showing the conflicts of interest of the 53 highly cited citations across 13 recommendation documents. APHA = American Public Health Association; NHMRC = National Health and Medical Research Council (AUS); NHS HS = NHS Health Scotland; NICE = National Institute for Health and Care Excellence (UK); PHAA = Public Health Association Australia; PHE = Public Health England; PHW = Public Health Wales; SGR = U.S. Department of Health and Human Services: A Report of the Surgeon General.

As shown in Fig 1 there are a large number of citations across one and two recommendation documents; 1508 (89% of 1700 citations) were cited by only one recommendation document. The number of citations per recommendation document varied (zero to 923 citations) meaning that documents with fewer citations provided less information to the citation network compared to documents with more citations. Only three recommendation documents cited over 100 citations and the U.S. Department of Health and Human Services: A Report of the Surgeon General (SGR) included 923 citations, more than double the number of citations in PHE 2018. There was a total of 53 citations across three or more recommendation documents (Fig 2). Of the 53 citations, we were interested in the study type (Fig 3) and the type of COI declared (Fig 4). COI were coded into five categories; details of each category are shown in S3 Table. Public Health Association Australia (PHAA), National Institute for Health and Care Excellence (NICE), and Public Health England (PHE) 2016 and 2019 were on the periphery of this network, sharing fewer citations in common than the other documents. SGR was most central, sharing citations with all other documents in the sample. NHS Health Scotland (2017) did not include any citations in the document and FDA does not cite any of the 53 influential citations therefore, these two documents are not shown in Figs 2–4.

The most common study type was non-epidemiology followed by systematic review (SR); with randomised control trial (RCT) being the least common.

Out of the 53 influential documents, 13 citations declared a COI, 14 made no explicit mention of COI, and 26 declared none (Fig 4). See S4 Table for the distribution of COI declared in 53 influential citations. We conducted further analysis on the 53 influential citations and found that the interpretation of several citations differed across the recommendation documents. For example, in relation to Hartmann-Boyce *et al*. [29], PHE 2018 stated e-cigarettes had a positive effect on smoking cessation. In contrast, Public Health Wales and National Health and Medical Research Council (NHMRC) stated that there were low levels of confidence in the study findings, but e-cigarettes were likely to help in smoking cessation. SGR stated “the majority of currently available scientific evidence does not support the recommendation to use e-cigarettes for the cessation of cigarette smoking” [30, p. 183] and the American Public Health Association (APHA) stated e-cigarettes “alone are not any more effective than other strategies” [31, p.9].

In addition, we analysed the 13 citations that declared a COI and found that the presence of COI was not explicitly taken into account when it was presented as evidence in any of the recommendation documents. The NHMRC document highlighted the importance of considering COI of authors when reviewing the evidence base.

### Blockmodelling

**Fig 5:**
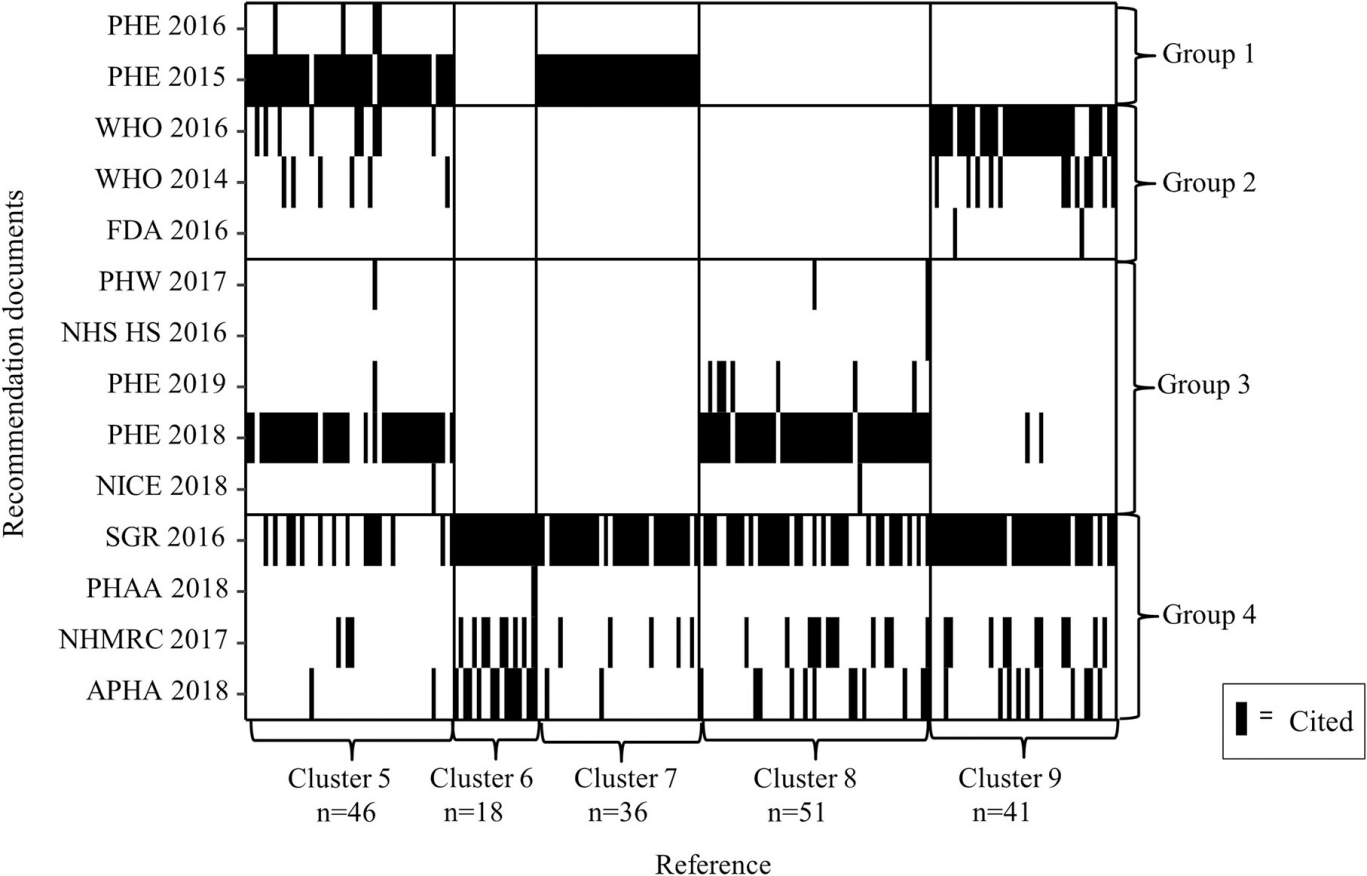
Clustering of recommendation documents by the number of shared references. APHA = American Public Health Association; FDA = U.S. Food and Drug Administration; NHMRC = National Health and Medical Research Council (AUS); NHS HS = NHS Health Scotland; NICE = National Institute for Health and Care Excellence (UK); PHAA = Public Health Association Australia; PHE = Public Health England; PHW = Public Health Wales; SGR = U.S. Department of Health and Human Services: A Report of the Surgeon General.

Blockmodelling was fitted to the network of citations included in more than one recommendation document, comprising 192 evidence sources (11% of 1700 citations). The best-fitting model contained four groups of recommendation documents (Groups 1–4) and five clusters of cited references (Clusters 5–9) (Fig 5). The log-likelihood for this model was -2601·83 and indicated a much better fit than fewer recommendation groups. There was a less clear differentiation between the number of clusters for evidence sources, with comparable likelihoods across a range of numbers of groups of recommendation documents (S5 Table for further details).

Each black rectangle in Fig 5 represents that a recommendation document (in the row) included a citation for the evidence source (in the columns, the citation labels are omitted for presentation). Where black lines appear in the same column on more than one row, this indicated that there was a common reference across the recommendation documents in those rows. Recommendation group 1 contained PHE 2015 and PHE 2016 documents, they drew on evidence from reference cluster 5 (n = 46) and 7 (n = 36). Recommendation group 2 contained the WHO documents and FDA, they exclusively drew on evidence from reference clusters 5 and 9 (n = 41). The recommendation documents in group 4 drew upon evidence clusters that were used by all other recommendation groups. This group corresponds with a central core of recommendation documents in Fig 2: SGR, NHMRC, and APHA. The evidence in cluster 5 was used by recommendation documents across all four groups. The evidence in cluster 8 (n = 51) was used exclusively by groups 3 and 4, corresponding to the more central recommendation documents in Fig 2: Public Health Wales, PHE 2018, SGR, NHMRC, and APHA. In summary, the blockmodelling uncovered elements of the literature that were common across all policy jurisdictions, as well as some that are distinct to different jurisdictions.

Investigating the distribution of COI statements across recommendation groups, we saw clear differences in proportions of evidence sources declaring or not declaring the presence or absence of COI (Table 3). Groups 2 and 4 are drawing on more conflicted declared articles than groups 1 and 3. Group 3 included fewer references not reporting COI statements and had low rates of declared COI. Results from the Fisher’s exact test indicated that these differences in distributions of COI are not random but represent clear distinctions in evidence used by recommendation documents. See S6 Table for the distribution of conflicts of interest per reference cluster.

**Table 3:** Distribution of conflicts of interest per recommendation block and results of the fisher’s exact test.

Recommendation group	Type of conflict			Fisher’s exact test
	None declared	No mention	Declared a COI	
**1**	76 (53·1%)	22 (15·4%)	45 (31·5%)	p = 0·02
**2**	30 (34·9%)	11 (12·8%)	45 (52·3%)	
**3**	40 (56·3%)	6 (8·5%)	25 (35·2%)	
**4**	36 (41·8%)	10 (11·6%)	40 (46·6%)	

### Conflicts of interest statements over time

**Fig 6:**
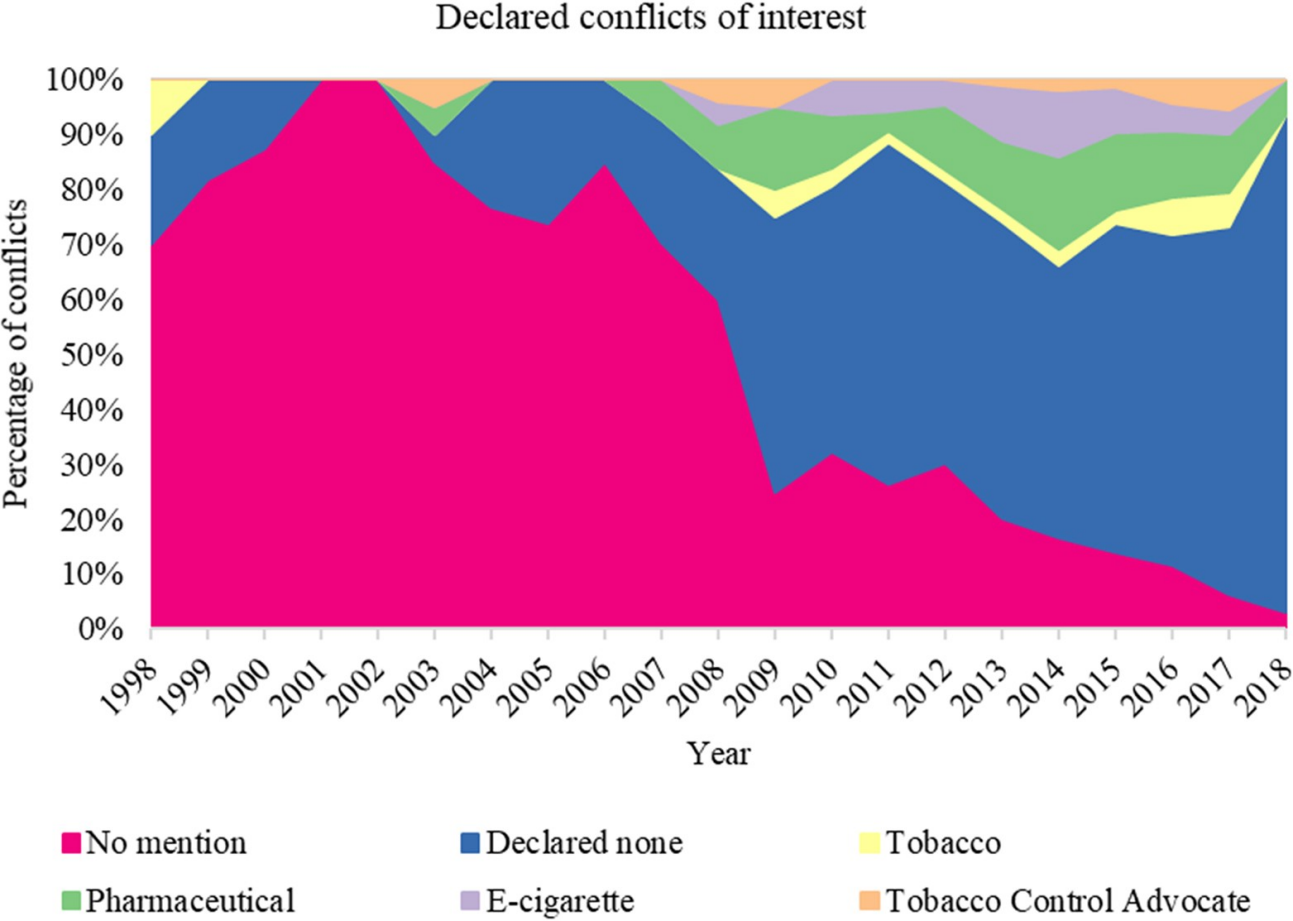
Percentage of declared conflicts of interest across citations between 1998–2018.

Of the 1179 cited journal articles 44 texts were unavailable and therefore were excluded from the analysis. Between 1965–1997 the majority of articles cited had no mention of COI, therefore, more detailed analyses of distributions of COI were restricted to publications post-1998. This demonstrates the reporting of COI has improved over time, as shown in Fig 6. Across the remaining journal articles (n = 1081), there was a total of 1142 declaration of COI (Fig 6). The number of declarations refers to individual authors, therefore there are multiple declarations within each article (See S3 Appendix for the number of journal articles published between 1998–2018).

Out of the 1081 journal articles published between 1998–2018, 288 contained no mention of COI, 593 declared no COI, and 261 declared COI. The reporting of COI has substantially improved over time, as shown in Fig 6. COI with e-cigarette companies first appear in 2008. The number increased over the next two years in 2012, 4% of articles declared COI with e-cigarette companies (2 articles of the n = 45 articles published), followed by 11% of articles 2013 (9 of n = 85), finally peaking in 2014 with 13% of articles (24 of n = 183). Following the peak in 2014, the number of articles that declared COI with e-cigarette companies reduced to 5% (5 of n = 106) in 2017 and zero (0 of n = 32) in 2018. COI associated with the tobacco industry were first visible in 1982. There is a consistent presence of tobacco company COI between 2009–2013, with the number increasing in 2015 to 3% of articles (5 articles of the n = 204) to 7% of articles (12 of n = 167). Overall, 3% of articles (37 of the 1081 articles published between 1998–2018) had tobacco, 7% (n = 72) e-cigarette, and 12% (n = 127) pharmaceutical COI.

## Discussion

Public health bodies across four jurisdictions vary in their approach to citing evidence to justify their recommendations, with some citing numerous sources whereas others not indicating the evidence used to develop their recommendations. There was some overlap in the sources of evidence drawn upon by public health bodies when making e-cigarette recommendations. However, this evidence was used to articulate different policy approaches; the UK adopting a ‘harm reduction’ approach and WHO, Australia, and USA adopting a ‘precautionary’ approach. The majority of the evidence cited was not shared across the recommendation documents, with relatively few influential citations.

Our analysis demonstrated that evidence influencing public health recommendation documents stems from research where COI are not declared or where important conflicts exist. A substantial proportion of cited evidence contained pharmaceutical (12%), e-cigarette (7%) or even tobacco (3%) COI, including amongst the most influential research featuring across multiple recommendation documents. While reporting of COI has substantially improved over time, there is still a substantial proportion of articles that do not explicitly report potential COI. The presence of COI associated with e-cigarette companies coincides with the introduction of e-cigarettes into the European and US market in 2006 and 2007, respectively. Several journals will no longer accept submissions of articles that have ties with the tobacco industry [32, 33]. Therefore, it is surprising to see the presence of COI involving tobacco companies as recently as 2017.

Several studies have investigated the relationship between industry COI and/or funding and research outcomes [13, 34–36]. Results from these studies demonstrated that funder interference is common across public health and can have an effect on the research agenda and can influence the results reported (e.g., reporting of industry favoured results). Miller *et al*. and Fabbri *et al*. argue that disclosure of COI and funding should be mandatory [34, 35]. Our study adds to the literature by demonstrating that the sources of evidence drawn upon by public health bodies when developing recommendation documents are subject to COI, including even the most concerning COI–funding from the tobacco industry.

Our study has several strengths. We systematically identified e-cigarette recommendation documents from four purposefully selected jurisdictions. We carried out a detailed investigation of the citations included in the 15 recommendation documents (with independent validation of data extraction). The use of citation network analysis to investigate and illustrate the sources of evidence drawn upon by public health bodies when making recommendations is a relatively novel method that highlights the inter-relationships between the evidence used by different public health bodies. However, some limitations should be noted. First, we examined only citations in relation to the types of research and COI rather than the quality of evidence and broader forms of evidence use. Second, it is highly likely that we are underestimating the presence of COI as we are reliant on what has been declared within each article and how COI are interpreted. Bindslev *et al*. and Rasmussen *et al*. showed that COI are often not declared [37, 38]. Research by Rasmussen *et al*. found that almost half of all authors had undisclosed COI in clinical trials [38]. Third, we are only examining citations and it is possible that in some cases citations may reflect critiques of presented evidence rather than evidence use. However, we conducted further analysis of the 53 most influential citations and found no examples of this. Fourth, we investigated the COI within journal articles and influential citations. Several of the citations analysed were SRs and it is worth noting that individual COI within the studies incorporated into the SRs are not included in declaration statements for the overall SR. Fifth, we examined only one case study (e-cigarettes) and there is a need for further research to investigate COI in recommendations for other public health areas. Finally, each of the recommendation documents included in the sample was produced at a specific time and to address slightly different remits. This is likely to lead to some divergence in the type and number of citations included, making comparison more challenging. Despite these limitations, this research draws on international data and investigates a priority for public health policy. Therefore, it is likely to be of interest to both policymakers and researchers internationally. Our findings about COI have important implications for public health policy, including highlighting a need for mechanisms to be implemented to guard against the undue influence of such COI. While we cannot establish that cited evidence which included COI definitively influenced decision-making, it is noteworthy that recommendation documents did not transparently record and consider COI in the underlying evidence base. Greater transparency in recommendation documents when drawing on evidence featuring COI may be warranted.

Our study highlights several areas of research that contribute to understanding the sources of evidence used in public health recommendations. There is a need to better understand the process used by different public health bodies when creating recommendations and how recommendation committees handle evidence where vested interests exist. To address this, an investigation of the views of those involved in the development of public health recommendations to explore the development process, the role of evidence, how COI are managed during the development process, and how contextual factors influence the development of recommendations. This would help to deepen our understanding of the development process and the role of evidence in public health recommendations. Our study was not able to determine and understand why different public health organisations have pursued different policy approaches based on the evidence. Further, more detailed analysis involving policy stakeholders may be required to understand this. The variation in the number of citations per recommendation document (e.g., six of 15 documents (40% of the sample) cited 11 or fewer citations from the total 1700 citations) impacted the visualisation of the citation network. The aim of this study was to investigate the sources of evidence used by public health bodies when making e-cigarette policy recommendations and COI within these sources rather than to investigate the quantity of citations included within a recommendation document. Future research could usefully explore how citations reflect the development of e-cigarette recommendations.

Public health recommendations aspire to be evidence-informed. Our study shows that the evidence relied upon when developing policy recommendations is subject to COI. The presence of COI could threaten the validity of the evidence base, therefore, shaping subsequent policy recommendations resulting in inappropriate public health actions. Using e-cigarettes as a case study we have demonstrated the need for robust methods to manage evidence derived from industry funding or incorporating industry COI within public health recommendations. These COI extend to even the most concerning industries, such as tobacco, and an urgent debate is needed about whether such evidence should inform public health policy.

## Supporting information

S1 Appendix PRISMA flow diagram and detailed search strategy for the sampling of documents. (DOCX)

S2 Appendix Retrieval of full text and conflicts of interest. (DOCX)

S3 Appendix Illustration of the number of journal articles published between 1998–2018. Distribution of the number of journal articles published between 1998–2018.
(DOCX)

S1 Table Year of publication and type of citation for all 1700 unique citations and number of conflicts of interest stated in 1135 accessible journal articles. (DOCX)

S2 Table Type of citation across the four jurisdictions. (DOCX)

S3 Table Definition of the five conflicts of interest categories and an example of each type. (DOCX)

S4 Table Distribution of conflicts of interest declared in 53 influential citations. (DOCX)

S5 Table Combinations of models identified for further analysis and corresponding log-likelihood. (DOCX)

S6 Table Distribution of conflicts of interest per reference cluster and results of the Fisher’s exact test. (DOCX)
