# Supplementary material for: Examining the sources of evidence in e-cigarette policy recommendations: A citation network analysis of international public health recommendations
Source: PLoS One. 2021 Aug 4;16(8):e0255604. doi: 10.1371/journal.pone.0255604 (PMC8336794; doi:10.1371/journal.pone.0255604)
Supplement: S1 Appendix — (DOCX) [file pone.0255604.s001.docx]

**S1 Appendix.** PRISMA flow diagram and detailed search strategy for the sampling of documents.


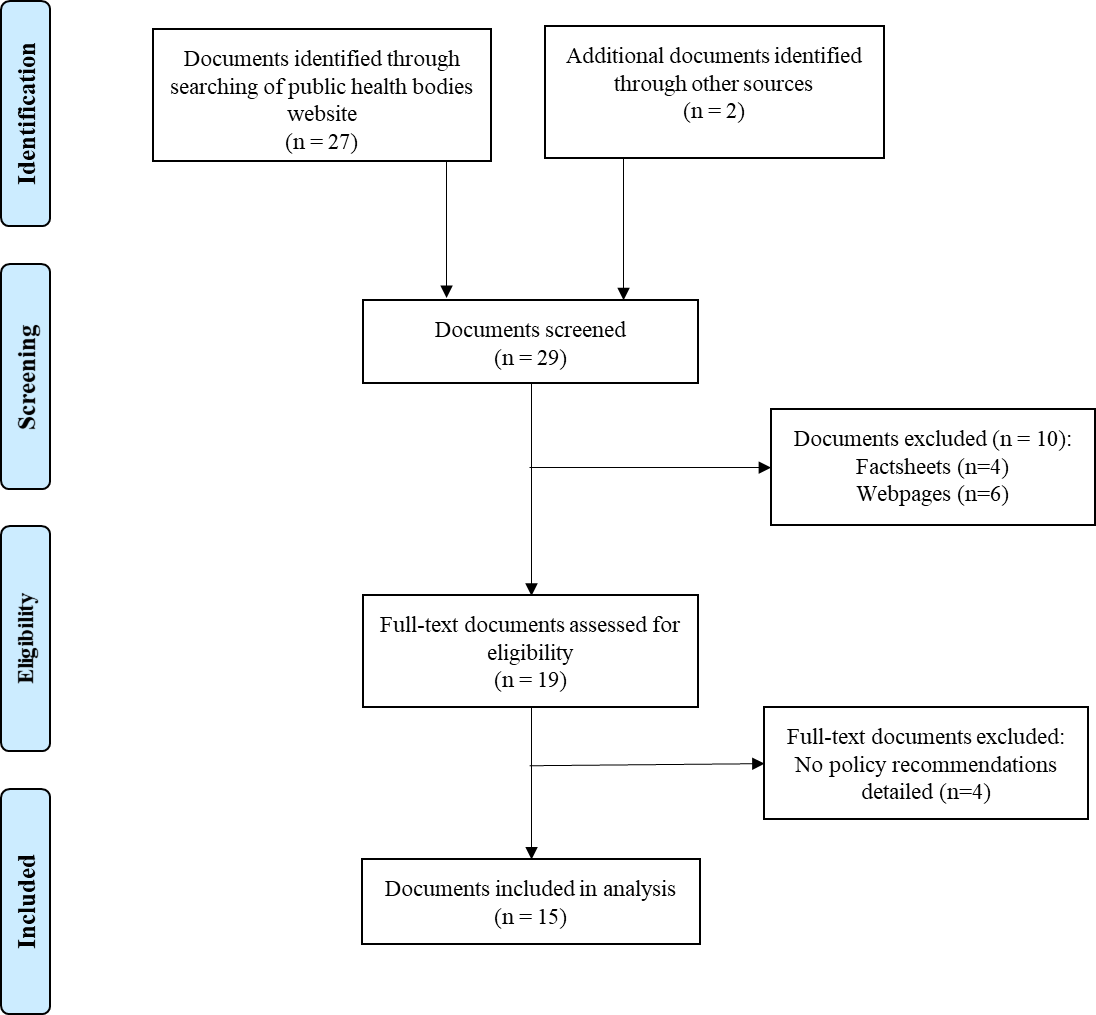


S1 Fig: PRISMA flow diagram.

1. **Records identified via keyword search of public health bodies website (n=27)**

**UK**

1. Public Health England: E-cigarettes: an evidence update (2015)
2. Public Health England: Use of e-cigarettes in public places and workplaces (2016)
3. Public Health England: Evidence review of e-cigarettes and heated tobacco products (2018)
4. Public Health England Vaping in England: an evidence update (2019)
5. The National Institute for Health and Care Excellence: Stop smoking intervention and services [NG92] (2018)
6. NHS Health Scotland: Smoke-free prisons and e-cigarettes (2016)
7. NHS Health Scotland: Consensus statement on e-cigarettes (2017)
8. Scottish Intercollegiate Guidelines Network: Risk Estimation and the prevention of cardiovascular disease: A national clinical guideline (2017)
9. Public Health Wales: E-cigarettes (Electronic Nicotine Delivery Systems (ENDS)) (2017)
10. Public Health Agency (Northern Ireland): Tobacco Control Northern Ireland (2015)

**Australia**

1. National Health and Medical Research Council NHMRC CEO Statement: Electronic Cigarettes (E-Cigarettes) (2017)
2. Public Health Association Australia: E-cigarettes policy position statement (2018)

**USA**

1. U.S. Department of Health and Human Services: E-Cigarette Use Among Youth and Young Adults: A Report of the Surgeon General (2016)
2. U.S. Department of Health and Human Services: Surgeon General’s Statement on FDA’s E-cigarette Prevention Campaign (2018)
3. U.S. Centre for Disease Control and Prevention: About Electronic Cigarettes (E-cigarettes) (ND)
4. U.S. Centre for Disease Control and Prevention: What’s the Bottom Line on the Risks of E-cigarettes for Kids, Teens, and Young Adults? (ND)
5. U.S. Centre for Disease Control and Prevention: E-cigarettes Talk to Youth about Risks (ND)
6. U.S. Centre for Disease Control and Prevention: E-cigarette, or vaping, products visual directory (ND)
7. U.S. Centre for Disease Control and Prevention: E-cigarettes shaped like USB flash drives (ND)
8. U.S. Food and Drug Administration: Deeming Tobacco Products To Be Subject to the Federal Food, Drug, and Cosmetic Act, as Amended by the Family Smoking Prevention and Tobacco Control Act; Restrictions on the Sale and Distribution of Tobacco Products and Required Warning Statements for Tobacco Products (2016)
9. U.S. Food and Drug Administration: How are Non-Combusted Cigarettes, Sometimes Called Heat-Not-Burn Products, Different from E-Cigarettes and Cigarettes? (ND)
10. National Institutes of Health- E-cigarettes summary (ND)
11. National Institutes of Health -Vaping Devices (Electronic Cigarettes) Drug Facts (ND)
12. National Institutes of Health -Tobacco, Nicotine, & Vaping (E-Cigarettes) (2020)
13. American Public Health Association: Supporting Regulation of Electronic Nicotine Delivery Systems (2018)

**WHO**

1. WHO: Electronic nicotine delivery systems (2014)
2. WHO: Electronic Nicotine Delivery Systems and Electronic Non-Nicotine Delivery Systems (ENDS/ENNDS) (2016)
3. **Additional documents identified through other sources (n=2)**
4. National Academy of Sciences, Engineering, and Medicine: Public Health consequences of e-cigarettes (2018)
5. U.S. Preventative Services Taskforce: Behavioural and Pharmacotherapy Interventions for Tobacco Smoking Cessation in Adults, Including Pregnant Women: U.S. Preventive Services Task Force Recommendation Statement (2015)
6. **Records screened (n=29)**
7. **Records excluded and why (n=10)**
8. U.S. Department of Health and Human Services: Surgeon General’s Statement on FDA’s E-cigarette Prevention Campaign (2018)- webpage/media release
9. U.S. Centre for Disease Control and Prevention: About Electronic Cigarettes (E-cigarettes) (ND)- webpage
10. U.S. Centre for Disease Control and Prevention: What’s the Bottom Line on the Risks of E-cigarettes for Kids, Teens, and Young Adults? (ND)- factsheet
11. U.S. Centre for Disease Control and Prevention: E-cigarettes Talk to Youth about Risks (ND)- factsheet
12. U.S. Centre for Disease Control and Prevention: E-cigarette, or vaping, products visual directory (ND)- factsheet
13. U.S. Centre for Disease Control and Prevention: E-cigarettes shaped like USB flash drives (ND)-factsheet
14. U.S. Food and Drug Administration: How are Non-Combusted Cigarettes, Sometimes Called Heat-Not-Burn Products, Different from E-Cigarettes and Cigarettes? (ND)- webpage
15. National Institutes of Health- E-cigarettes summary (ND) -webpage
16. National Institutes of Health -Vaping Devices (Electronic Cigarettes) Drug Facts (ND)- webpage
17. National Institutes of Health -Tobacco, Nicotine, & Vaping (E-Cigarettes) (2020) -webpage
18. **Full-text records assessed for eligibility (n=19)**
19. **Full-text records excluded and why (n=4)**
20. Scottish Intercollegiate Guidelines Network: Risk Estimation and the prevention of cardiovascular disease: A national clinical guideline (2017)- No recommendations on policy, only research recommendations.
21. Public Health Agency (Northern Ireland): Tobacco Control Northern Ireland (2015)- No recommendations on policy
22. National Academy of Sciences, Engineering, and Medicine: Public Health consequences of e-cigarettes (2018)- Provides research recommendations not policy
23. U.S. Preventative Services Taskforce: Behavioural and Pharmacotherapy Interventions for Tobacco Smoking Cessation in Adults, Including Pregnant Women: U.S. Preventive Services Task Force Recommendation Statement (2015) – No policy recommendations
24. **Records included in the analysis (n=15)**

**UK (n=8)**

1. Public Health England: E-cigarettes: an evidence update (2015)
2. Public Health England: Use of e-cigarettes in public places and workplaces (2016)
3. Public Health England: Evidence review of e-cigarettes and heated tobacco products (2018)
4. Public Health England: Vaping in England: an evidence update (2019)
5. The National Institute for Health and Care Excellence: Stop smoking intervention and services [NG92] (2018)
6. NHS Health Scotland: Smoke-free prisons and e-cigarettes (2016)
7. NHS Health Scotland: Consensus statement on e-cigarettes (2017)
8. Public Health Wales: E-cigarettes (Electronic Nicotine Delivery Systems (ENDS)) (2017)

**Australia (n=2)**

1. National Health and Medical Research Council NHMRC CEO Statement: Electronic Cigarettes (E-Cigarettes) (2017)
2. Public Health Association Australia – E-cigarettes policy position statement (2018)

**USA (n=3)**

1. U.S. Department of Health and Human Services: E-Cigarette Use Among Youth and Young Adults: A Report of the Surgeon General (2016)
2. U.S. Food and Drug Administration: Deeming Tobacco Products To Be Subject to the Federal Food, Drug, and Cosmetic Act, as Amended by the Family Smoking Prevention and Tobacco Control Act; Restrictions on the Sale and Distribution of Tobacco Products and Required Warning Statements for Tobacco Products (2016)
3. American Public Health Association: Supporting Regulation of Electronic Nicotine Delivery Systems (2018)

**WHO (n=2)**

1. WHO: Electronic nicotine delivery systems (2014)
2. WHO: Electronic Nicotine Delivery Systems and Electronic Non-Nicotine Delivery Systems (ENDS/ENNDS) (2016)
