# Supplementary material for: Examining the sources of evidence in e-cigarette policy recommendations: A citation network analysis of international public health recommendations
Source: PLoS One. 2021 Aug 4;16(8):e0255604. doi: 10.1371/journal.pone.0255604 (PMC8336794; doi:10.1371/journal.pone.0255604)
Supplement: S2 Appendix — (DOCX) [file pone.0255604.s002.docx]

**S2 Appendix.** Retrieval of full text and conflicts of interest

To find cited texts, we constructed a Shiny app (S2 Fig) to display each citation, extract author and title, record publication type, and search SCOPUS to identify journal-published articles. We retrieved full texts for each available article and extracted the phrases “Funding”, “Interests”, “Conflict*” and “Declar*”, with the 100 characters preceding it and the 300 characters following. We then imported the articles with search results into a second Shiny app (S2 Fig) to read and code for the presence or absence of COI and the types of COI present. The 1491 articles were coded by MS, with 10% of that doubled-checked by AB. We grouped COI statements by type and plotted to show densities of types by year of publication. We then retrieved full texts, categorised each by publication type, and extracted declared COI.


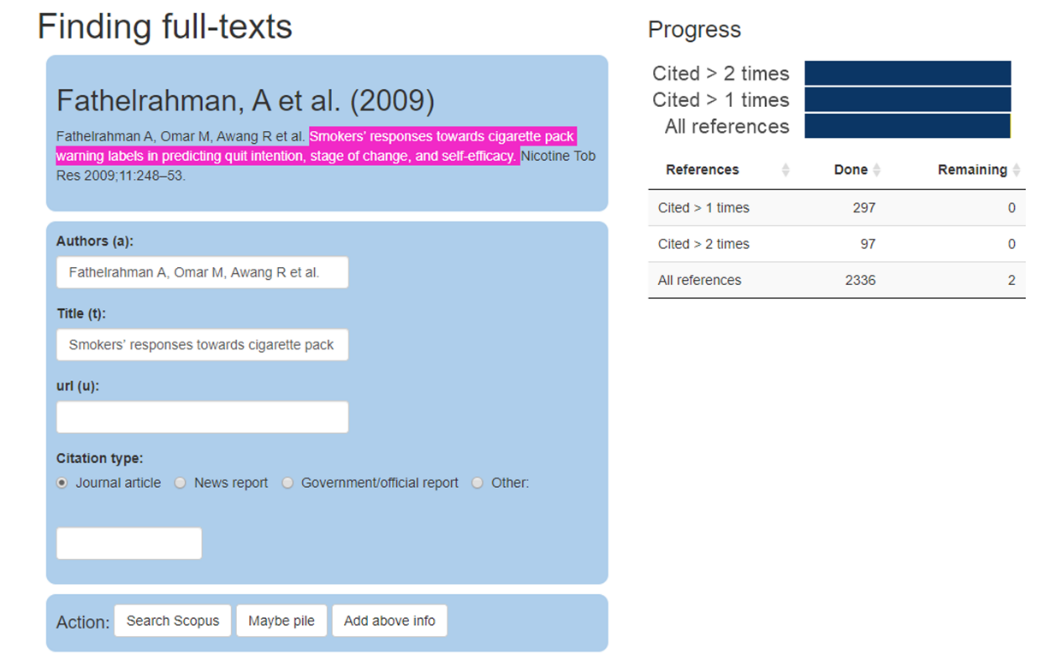


S2 Fig: Screenshot of the Shiny app used to find the full text of all journal articles.


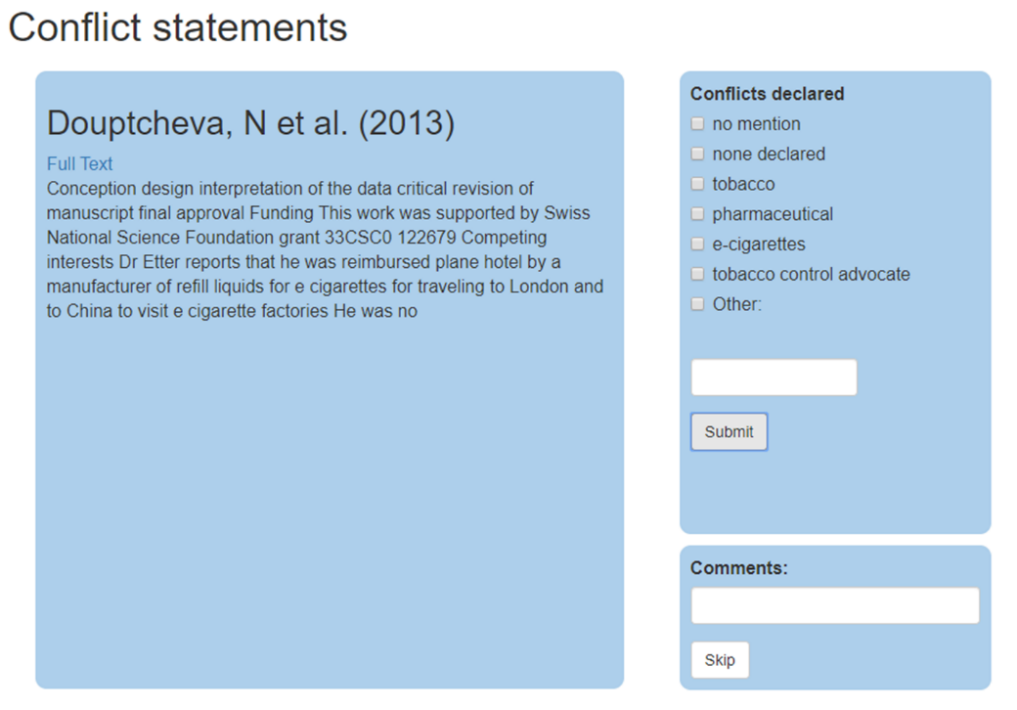


S3 Fig: Screenshot of the Shiny app used to extract conflicts of interest.
