# Supplementary material for: Examining the sources of evidence in e-cigarette policy recommendations: A citation network analysis of international public health recommendations
Source: PLoS One. 2021 Aug 4;16(8):e0255604. doi: 10.1371/journal.pone.0255604 (PMC8336794; doi:10.1371/journal.pone.0255604)
Supplement: S3 Appendix — Distribution of the number of journal articles published between 1998–2018. (DOCX) [file pone.0255604.s003.docx]

**S3 Appendix.** Illustration of the number of journal articles published between 1998-2018.


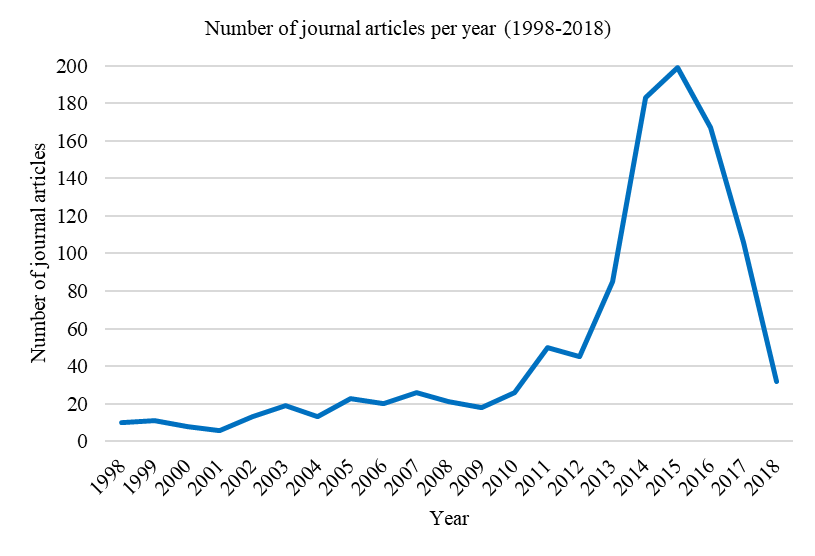


S4 Fig: Distribution of the number of journal articles published between 1998-2018.
