# Supplementary material for: Examining the sources of evidence in e-cigarette policy recommendations: A citation network analysis of international public health recommendations
Source: PLoS One. 2021 Aug 4;16(8):e0255604. doi: 10.1371/journal.pone.0255604 (PMC8336794; doi:10.1371/journal.pone.0255604)
Supplement: S1 Table — (DOCX) [file pone.0255604.s004.docx]

**S1 Table.** Year of publication and type of citation for all 1700 unique citations and number of conflicts of interest stated in 1135 accessible journal articles.

| **Year of publication** | **Number of studies (n=1700)** |
| --- | --- |
| Pre 1990 | 39 (2·3%) |
| 1990-1999 | 75 (4·4%) |
| 2000-2009 | 230 (13·5%) |
| 2010 onwards | 1331 (78·3%) |
| No date | 25 (1·5%) |
| **Type of citation** |  |
| Book | 15 (0·9%) |
| Comment | 1 (0·1%) |
| Conference proceedings | 11 (0·6%) |
| E-cigarette company | 3 (0·2%) |
| Government/official report | 267 (15·7%) |
| Journal article | 1179 (69·4%) |
| News report | 72 (4·2%) |
| Other | 4 (0·2%) |
| Policy think tank | 4 (0·2%) |
| Public health website | 39 (2·3%) |
| Social media | 5 (0·3%) |
| Statistical report | 85 (5·0%) |
| Tobacco company | 15 (0·9%) |
| **Types of conflict of interest** | **Number of declarations (across 1135 accessible journal articles)** |
| Declared none | 326 (27·6%) |
| No mention | 594 (50·2%) |
| Pharmaceutical | 38 (3·2%) |
| E-cigarette | 128 (10·8%) |
| Tobacco company | 72 (6·1%) |
| Tobacco control advocate | 25 (2·1%) |
