# Supplementary material for: Examining the sources of evidence in e-cigarette policy recommendations: A citation network analysis of international public health recommendations
Source: PLoS One. 2021 Aug 4;16(8):e0255604. doi: 10.1371/journal.pone.0255604 (PMC8336794; doi:10.1371/journal.pone.0255604)
Supplement: S2 Table — (DOCX) [file pone.0255604.s005.docx]

**S2 Table.** Type of citation across the four jurisdictions.

| **Type of citation** | **Number of citations** | Subset cited across jurisdictions | | | |
| --- | --- | --- | --- | --- | --- |
|  |  | **WHO** | **UK** | **Australia** | **USA** |
| Book | 15 | 0 | 0 | 0 | 15 |
| Comment | 1 | 0 | 0 | 0 | 1 |
| Conference proceedings | 11 | 0 | 9 | 1 | 1 |
| E-cigarette company press release | 3 | 0 | 0 | 0 | 3 |
| Government/official report | 267 | 15 | 104 | 11 | 154 |
| Journal article | 1179 | 81 | 458 | 60 | 744 |
| News report | 72 | 6 | 14 | 0 | 52 |
| Other | 4 | 0 | 1 | 0 | 3 |
| Policy think tank | 4 | 1 | 0 | 0 | 3 |
| Public health website | 39 | 1 | 20 | 1 | 17 |
| Social media | 5 | 1 | 1 | 0 | 3 |
| Statistical report | 85 | 3 | 37 | 1 | 48 |
| Tobacco company | 15 | 4 | 3 | 0 | 8 |
| Total | 1700 | 112 | 647 | 74 | 1,032 |
