# Supplementary material for: Examining the sources of evidence in e-cigarette policy recommendations: A citation network analysis of international public health recommendations
Source: PLoS One. 2021 Aug 4;16(8):e0255604. doi: 10.1371/journal.pone.0255604 (PMC8336794; doi:10.1371/journal.pone.0255604)
Supplement: S3 Table — (DOCX) [file pone.0255604.s006.docx]

**S3 Table.** Definition of the five conflicts of interest categories and an example of each type.

| **Type of conflict of interest** | **Definition** | **Example from literature** |
| --- | --- | --- |
| Declared none | Authors declared no conflicts of interest. | *“The authors have no conflict of interest to declare.”[1]* |
| No mention | There was no mention of authors’ conflicts of interest by authors | N/A |
| Pharmaceutical | Study was fully or partially funded by a pharmaceutical company and/or authors had received financial payments from companies for consulting, advisory roles, speaking, travel expenses etc from the pharmaceutical industry. | *“MLG received a research grant from Pfizer and served as an advisory board member to Johnson Johnson manufacturers of smoking cessation medications.”[2]* |
| Both e-cigarette and pharmaceutical | Study was fully or partially funded by a pharmaceutical company and e-cigarette company and/or authors had received financial payments from companies for consulting, advisory roles, speaking, travel expenses etc from the pharmaceutical industry and e-cigarette industry. | *“MLG received research funding from Pfizer, manufacturer of stop smoking medication, and was funded by the UK Centre for Tobacco Control Studies (UKCTCS) during the study. AS received research funds and travel expenses from Chic Group Ltd., manufacturer of electronic cigarettes in Poland. Other authors declare no conflicts of interest.”[3]* |
| E-cigarette | Study was fully or partially funded by e-cigarette manufacture and/or authors had received financial payments from companies for consulting, advisory roles, speaking, travel expenses etc from the e-cigarette industry. | *This study was conducted in Celerion (Lincoln, Nebraska), funded by the LOEC, Inc. d/b/a blu ecigs”[4]* |

**References**

1. Behar RZ, Davis B, Wang Y, Bahl V, Lin S, Talbot P. Identification of toxicants in cinnamon-flavored electronic cigarette refill fluids. Toxicology in Vitro. 2014;28(2):198-208.

2. Levy DT, Borland R, Lindblom EN, Goniewicz ML, Meza R, Holford TR, et al. Potential deaths averted in USA by replacing cigarettes with e-cigarettes. Tobacco Control. 2018;27(1):18.

3. Czogala J, Goniewicz ML, Fidelus B, Zielinska-Danch W, Travers MJ, Sobczak A. Secondhand exposure to vapors from electronic cigarettes. Nicotine & tobacco research : official journal of the Society for Research on Nicotine and Tobacco. 2014;16(6):655-62.

4. Yan XS, D’Ruiz C. Effects of using electronic cigarettes on nicotine delivery and cardiovascular function in comparison with regular cigarettes. Regulatory Toxicology and Pharmacology. 2015;71(1):24-34.
