# Supplementary material for: Examining the sources of evidence in e-cigarette policy recommendations: A citation network analysis of international public health recommendations
Source: PLoS One. 2021 Aug 4;16(8):e0255604. doi: 10.1371/journal.pone.0255604 (PMC8336794; doi:10.1371/journal.pone.0255604)
Supplement: S4 Table — (DOCX) [file pone.0255604.s007.docx]

**S4 Table. Distribution of conflicts of interest declared in 53 influential citations.**

| **Jurisdiction**  **Organisation** | | **Not containing COI** | | **Containing COI** | | | **Total** | **Proportion with COI** |
| --- | --- | --- | --- | --- | --- | --- | --- | --- |
|  |  | **None declared** | **No mention** | **Pharmaceutical** | **Both e-cigarette and pharmaceutical** | **E-cigarette** |  |  |
| **International** | WHO | 20 | 7 | 4 | 5 | 0 | **36** | **25%** |
| **UK** | NHS HS | 0 | 1 | 0 | 0 | 0 | **1** | **0%** |
|  | NICE | 0 | 0 | 0 | 1 | 0 | **1** | **100%** |
|  | PHE | 21 | 13 | 10 | 9 | 2 | **55** | **38%** |
|  | PHW | 0 | 1 | 1 | 1 | 0 | **3** | **67%** |
| **Australia** | NHRMC | 12 | 8 | 1 | 2 | 0 | **23** | **13%** |
|  | PHAA | 0 | 1 | 0 | 0 | 0 | **1** | **0%** |
| **USA** | APHA | 11 | 4 | 0 | 2 | 0 | **17** | **12%** |
|  | FDA | 0 | 0 | 0 | 0 | 0 | **0** | **0%** |
|  | SGR | 21 | 14 | 3 | 6 | 1 | **45** | **22%** |

APHA=American Public Health Association

FDA=U.S. Food and Drug Administration

NHMRC=National Health and Medical Research Council (AUS)

NHS HS=NHS Health Scotland

NICE=National Institute for Health and Care Excellence (UK)

PHAA=Public Health Association Australia

PHE=Public Health England

PHW=Public Health Wales

SGR=U.S. Department of Health and Human Services: A Report of the Surgeon General
