# Supplementary material for: Examining the sources of evidence in e-cigarette policy recommendations: A citation network analysis of international public health recommendations
Source: PLoS One. 2021 Aug 4;16(8):e0255604. doi: 10.1371/journal.pone.0255604 (PMC8336794; doi:10.1371/journal.pone.0255604)
Supplement: S5 Table — (DOCX) [file pone.0255604.s008.docx]

**S5 Table.** Combinations of models identified for further analysis and corresponding log-likelihood.

Blockmodelling was fit to the network of citations cited in more than one recommendation document. We identified four possible models for further analysis. The best-fitting model contained five clusters of cited references and four groups of recommendation documents and produced a relatively good fit whilst maintaining a few blocks, with all blocks containing more than one unit.

| **Reference (x)** | **Recommendation document (y)** | **Log-likelihood** |
| --- | --- | --- |
| 3 | 3 | -2664·26 |
| 4 | 4 | -2626·90 |
| 5 | 4 | -2601·83 |
| 6 | 6 | -2559·27 |
