# Supplementary material for: Examining the sources of evidence in e-cigarette policy recommendations: A citation network analysis of international public health recommendations
Source: PLoS One. 2021 Aug 4;16(8):e0255604. doi: 10.1371/journal.pone.0255604 (PMC8336794; doi:10.1371/journal.pone.0255604)
Supplement: S6 Table — (DOCX) [file pone.0255604.s009.docx]

**S6 Table.** Distribution of conflicts of interest per reference cluster and results of the Fisher’s exact test.

In addition to examining the variation of COI across the recommendation groups, we also found distinct patterns of distribution of COI across reference clusters. Clusters 6 and 7 have up to twice as many COI (and lower proportions of ‘No mention’) compared to the other clusters. Results from the Fisher’s exact test indicates that these differences in distributions of COI are not due to random chance but represent clear distinctions in evidence used by recommendation documents.

| **Reference cluster** | **Type of COI** | | | **Fisher’s exact test** |
| --- | --- | --- | --- | --- |
|  | **None declared** | **No mention** | **Declared a COI** |  |
| **5** | 43 (45·7%) | 17 (18·1%) | 34 (36·2%) | p=0·00050 |
| **6** | 28 (37·8%) | 2 (2·7%) | 44 (59·5%) |  |
| **7** | 13 (27·1%) | 4 (8·3%) | 31 (64·6%) |  |
| **8** | 40 (48·8%) | 16 (19·5%) | 26 (31·7%) |  |
| **9** | 58 (65·9%) | 10 (11·4%) | 20 (22·7%) |  |
